# What Is the Place of Intersphincteric Resection When Operating on Low Rectal Cancer?

**DOI:** 10.5402/2012/585484

**Published:** 2012-08-01

**Authors:** Satoshi Nagayama, Waheeb Al-Kubati, Yoshiharu Sakai

**Affiliations:** ^1^Department of Gastroenterological Surgery, Cancer Institute Hospital, Tokyo 135-8550, Japan; ^2^Department of Colorectal Surgery, Concord Hospital, Sydney, NSW 2139, Australia; ^3^Department of Surgery, Kyoto University Hospital, Kyoto 606-8507, Japan

## Abstract

Operating on low rectal cancer by performing an intersphincteric resection (ISR) with coloanal anastomosis has been adopted as an alternative to abdominoperineal excision (APE) following Schiessel et al. report in 1994, as it preserves the sphincter and avoids the need for a permanent stoma. We undertook a review of the recent literature specifically focusing on long-term oncologic and functional outcomes of ISR to evaluate whether this operation is a valid alternative to an APE. In conclusion, younger patients with T1 or T2 rectal cancers who require no preoperative therapy are ideal candidates for ISR, given that preoperative chemoradiotherapy may cause long-term severe anal dysfunction after ISR.

An intersphincteric resection (ISR) with coloanal anastomosis for low rectal cancers has been adopted as an alternative to abdominoperineal excision (APE) after the report by Schiessel et al. in 1994, who succeeded in preserving the sphincter and avoiding the need for a permanent stoma [1]. 

In ISR, the rectum is mobilized to the levator ani muscle in the plane of total mesorectal excision (TME) via the abdominal route and the internal anal sphincter (IAS) is resected via the anal route. There are three types of ISR (partial, subtotal, or total) depending on the distal resection line of the IAS or the extent of the IAS resection (Figure 1). Coloanal anastomosis is performed using a transanal hand-sewn technique. An ultralow, anterior resection of the rectum using a double-stapling technique is not regarded as an ISR. 

An extensive review of 21 studies concerning 612 patients showed a local recurrence (LR) after ISR of 9.5%, and an average five-year overall survival (OS) rate of 81.5% [2]. In most of these studies a significant reduction in resting anal pressure was evident after surgery, but the same was not true of squeeze pressure. These authors suggest that ISR should be considered as an option for sphincter-preserving surgery when operating on patients with low rectal cancers and that it has acceptable oncologic and functional results. 

We decided to undertake a review of the recent literature specifically focusing on long-term oncologic and functional outcomes of ISR to evaluate whether this operation is a valid alternative to an APE. 

Yamada et al. [3] reviewed 107 patients who underwent ISR for low rectal cancer (partial ISR 69; subtotal ISR 16; total ISR 19) describing the long-term functional and oncologic results in patients where the distal margin of tumours was located within 35 mm of the dentate line; where there was no invasion of the external anal sphincter or levator ani muscle; where none had received preoperative treatment; where a distal clearance of at least 2 cm was possible for T2 and T3 tumours and 1 cm for T1 tumours. They reported a postoperative morbidity of 25.0% for patients undergoing a total ISR, 18.8% in those undergoing a subtotal ISR, and 11.3% for those undergoing a partial ISR. No patients required reoperation for complications related to their surgery. The five-year disease-free survival (DFS) rates were 100%, 83.5%, and 72.0% for TNM stages I, II, and III, respectively. The five-year cumulative LR rate was low at 2.5%. Concerning the functional outcome, 11 of the 19 patients (57.9%) who had a total ISR, 10 of the 16 (62.5%) who underwent a subtotal ISR, and 54 of the 69 (78.3%) who underwent a partial ISR demonstrated satisfactory faecal continence, assessed using Kirwan grading [4]. Multivariate analysis showed that the patient's age at surgery was the only factor associated with a risk of faecal incontinence. The authors concluded that total ISR may be the optimal sphincter-preserving surgery for patients with low rectal cancer, excluding elderly patients or patients with known preexisting functional impairment.

The long-term oncologic outcomes have also been analysed retrospectively for 202 consecutive patients who underwent curative ISR (132) or APE (70) for low-lying, primary rectal cancers located between 1 to 5 cm from the anal verge [5]. LR including regional lymph node metastases occurred in 14 patients (10.6%) from the ISR group and in 11 patients (15.7%) from the APE group. The five-year LR-free survival rates were not significantly different between the two groups. In addition, the five-year DFS rates were 69.1% in the ISR group and 63.3% in the APE group. In this study the conclusion was that ISR provided acceptable oncological results, since the LR and DFS rates were not compromised after ISR when compared to patients who had had an APE.

In a study of 120 consecutive patients with T1–T3 rectal cancers located 1.0 to 5.0 cm from the anal verge who did not receive preoperative chemoradiotherapy (CRT) [6], the three-year rates for distant metastasis and LR were 13% and 6%, respectively. Concerning distant metastases, there were no risk factors associated with the surgical procedure of ISR. In contrast, the resection margin and the degree of tumour differentiation were significantly associated with LR, although neither the T-stage nor postoperative anastomotic leaks were risk factors for LR. Akasu et al. [6] suggest that ISR may be performed for patients with T1-T2 rectal cancers who do not need preoperative treatment, as they acknowledge that preoperative CRT is superior in controlling LR though it may cause severe anal dysfunction postoperatively. 

Postoperative anal sphincter function was evaluated in 96 patients with very low rectal cancers who underwent partial (27), subtotal (43), and total (26) ISRs with a diverting stoma by questionnaires at 3, 6, 12, and 24 months after stomal closure [7]. Both anal dysfunction and incontinence scores assessed by both Wexner and Kirwan grading were significantly improved at 24 months postoperatively as compared to three months postoperatively. These results suggested that anal function may improve gradually to a satisfactory level, even if anal dysfunction is evident immediately following stoma closure. However, the authors [7] found that postoperative anal function of the 40 patients who had received preoperative CRT was severely impaired, regardless of the type of ISR. In addition, multivariate analysis demonstrated that preoperative CRT was the only independent factor associated with poor anal function after ISR, which was consistent with a prior study on the long-term functional results [8]. It would seem therefore that in those patients who require preoperative CRT, one should take into consideration the possibility that ISR may result in poor anal function even if the oncologic outcomes are regarded as acceptable. 

Weiser et al. [9] in their retrospective study investigated the oncologic outcomes of sphincter-preserving surgery in 148 patients with T3-T4 rectal cancers located less than 6 cm from the anal verge and treated by preoperative CRT followed by TME. Eighty-five (57%) of these patients had a sphincter-preserving resection. Of these 41 had a low anterior resection (LAR), 44 had an ISR, and 63 patients had an APE. There were a total of seven LRs: 1, 0, and 6 in the LAR, ISR and APE groups, respectively. The five-year recurrence-free survival rates for the LAR, ISR, and APE groups were estimated at 85%, 83%, and 47%, respectively. The five-year disease-specific survival rates for the LAR, ISR, and APE groups were 97%, 96%, and 59%, respectively. These authors concluded that sphincter preservation was feasible by combining preoperative CRT and ISR without compromising the oncologic outcomes. However, even when a complete response to CRT is demonstrated viable cancer cells may remain in the tumour bed. Further study therefore is needed to evaluate whether the choice of ISR for highly selected patients with very low rectal cancers would be acceptable oncologically, based on the post-CRT response, particularly when an APE would normally be performed without prior adjuvant therapy. In addition, prospective analyses showing acceptable anal function and quality of life following ISR combined with preoperative CRT are required before this procedure will be more widely adopted. Most recently Fujimoto et al. [10] reported favourable short-term postoperative outcomes in 35 patients with very low rectal cancers who underwent laparoscopic ISR and in the future ISR performed laparoscopically may be the preferred sphincter-preserving procedure.

One could conclude then that younger patients with T1 or T2 rectal cancers who require no preoperative therapy are ideal candidates for ISR, given that preoperative CRT may cause long-term severe anal dysfunction after ISR.

## Figures and Tables

**Figure 1:**
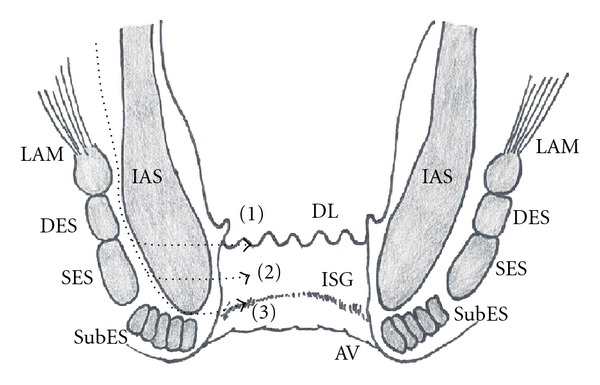
Schematic representation of the transaction lines for intersphincteric resection (ISR). The distal resection line of the internal anal sphincter (IAS) was at the dentate line (DL) (1) in partial ISR, between the DL and the intersphincteric groove (ISG) (2) in subtotal ISR, and at the ISG (3) in total ISR. AV: anal verge; DES: deep part of external sphincter; SES: superficial part of external sphincter; SubES: subcutaneous part of external sphincter; LAM: levator ani muscle.
